# Supplementary material for: Stroke risk associated with NSAIDs uses in women with dysmenorrhea: A population-based cohort study
Source: PLoS One. 2021 Nov 12;16(11):e0259047. doi: 10.1371/journal.pone.0259047 (PMC8589167; doi:10.1371/journal.pone.0259047)
Supplement: S1 Appendix — (DOCX) [file pone.0259047.s001.docx]

**S1 Appendix: Disease ICD-9 Code**

| Disease | ICD-9-CM |
| --- | --- |
| Stroke | 430-437 |
| Diabetes | 250 |
| dysmenorrhea | 625.3 |
| Hypertension | 401-405 |
| Hyperlipidemia | 272 |
| Obesity | 278 |
| Arrhythmia | 427 |
| Thyroid disease | 240-246 |
| Alcohol-related diseases | 291, 3030, 305, 571.0-571.3, 790.3, V11.3 |

Ref: The International Classification of Diseases, 9th Revision
