# Supplementary material for: Stroke risk associated with NSAIDs uses in women with dysmenorrhea: A population-based cohort study
Source: PLoS One. 2021 Nov 12;16(11):e0259047. doi: 10.1371/journal.pone.0259047 (PMC8589167; doi:10.1371/journal.pone.0259047)
Supplement: Attachment — Submitted filename: Stroke Risk associated with NSAIDs Uses in Women with Dysmenorrhea.docx [file pone.0259047.s002.docx]

Stroke Risk associated with NSAIDs Uses in Women with Dysmenorrhea;a population-based Cohort Study

Recommendations

1. Good article with a lot of data presented in the manuscript.
2. Abstract: results section: statement is too generalised. Suggest to reword to highlight the important results
3. Abstract: conclusion does not reflect the objective of the study, consider to emphasize the main ideas
4. Page 8 : Statement ‘ the study results indicated…..’ suggest to put in the discussion
5. Figure 1 comparing 70(0.32%) controls vs 27 (0.83%) cases. Please indicate the p value
6. The authors suggest that NSAIDs have a high risk for stroke. However, the table 1 showed s those on NSAIDs also have other comorbidities (diabetes, hypertension etc) which are the traditional risk factors for stroke. How do the authors taken this into account? Arrhythmia, thyroid disease, hyperlipidemia accounted for the high number of cases. However, need to specify if it was atrial fibrillation as this will cause high incidence of embolic stroke
7. Authors did not specify what type of stroke in the article? Infarct or haemorrhage ? How was the diagnosis of stroke made?
8. In the text, certain words like ‘women with dysmenorrhea’ are in upper case. Please change to lower case.
9. Discussion should highlight on the study’s main findings regarding NSAIDs use and stroke risk
10. Table 2 need to simplify to allow easy understanding
11. Table 3 – less than 5, use the symbol
12. References are not standardised. Books and journals, 7,8,17,20,21,23,24,25,27,28,30,31
