# Supplementary material for: Stroke risk associated with NSAIDs uses in women with dysmenorrhea: A population-based cohort study
Source: PLoS One. 2021 Nov 12;16(11):e0259047. doi: 10.1371/journal.pone.0259047 (PMC8589167; doi:10.1371/journal.pone.0259047)
Supplement: Attachment — Submitted filename: 20210821_reponse to reviewer.docx [file pone.0259047.s003.docx]

**Response to Reviewers’ comments**

**Manuscript ID:** [PONE-D-21-13819] - [EMID:95d7e86a2c12ef75]

**MS TITLE:** Stroke Risk associated with NSAIDs Uses in Women with Dysmenorrhea: a population-based Cohort Study

The authors thank the reviewers for their helpful and thoughtful comments and suggestions. In the document below we have listed our responses point by point. Reviewers’ comments are numbered and in Cambria font. Responses follow in Calibri font.

**Response to Editor**

**Editor Comments to the Author**

**1.** *Please ensure that your manuscript meets PLOS ONE's style requirements, including those for file naming. The PLOS ONE style templates can be found at*

*https://journals.plos.org/plosone/s/file?id=wjVg/PLOSOne_formatting_sample_main_body.pdf and*

*https://journals.plos.org/plosone/s/file?id=ba62/PLOSOne_formatting_sample_title_authors_affiliations.pdf*

**Reply:** Thank you for the comments and suggestion. We have revised the manuscript to ensure that our manuscript meets the publication style of PLOS ONE.

(Please see the revised manuscript.)

**2.** *Thank you for including your ethics statement: "the database was approved by the Research Ethics Committee of China Medical University and Hospital, Taichung, Taiwan (CMUH104-REC2-115)".*

*Please provide additional details regarding participant consent. In the ethics statement in the Methods and online submission information, please ensure that you have specified (1) whether consent was informed and (2) what type you obtained (for instance, written or verbal, and if verbal, how it was documented and witnessed). If your study included minors, state whether you obtained consent from parents or guardians. If the need for consent was waived by the ethics committee, please include this information.*

*If you are reporting a retrospective study of medical records or archived samples, please ensure that you have discussed whether all data were fully anonymized before you accessed them and/or whether the IRB or ethics committee waived the requirement for informed consent. If patients provided informed written consent to have data from their medical records used in research, please include this information.*

*Once you have amended this/these statement(s) in the Methods section of the manuscript, please add the same text to the “Ethics Statement” field of the submission form (via “Edit Submission”).*

*For additional information about PLOS ONE ethical requirements for human subjects research, please refer to http://journals.plos.org/plosone/s/submission-guidelines#loc-human-subjects-research.*

**Reply:** Thank you for the comment and suggestions. To protect the patients’ privacy, all personal identification numbers were encrypted by the National Health Insurance before the data were released. The National Health Insurance Research Database (NHIRD) encrypts patient personal information to protect privacy and provides researchers with anonymous identification numbers associated with relevant claims information, including sex, date of birth, medical services received, and prescriptions. Therefore, patient consent is not required to access the NHIRD. This study was approved to fulfill the condition for exemption by the Institutional Review Board (IRB) of China Medical University (CMUH-104-REC2-115-CR4). The IRB also specifically waived the consent requirement.

***3.*** *Thank you for stating the following in the Acknowledgments Section of your manuscript:*

**Reply:** "This study was supported in part by grants from the Ministry of Health and Welfare, Taiwan (MOHW108-TDU-B-212-133004), China Medical University Hospital (DMR-108-024 and DMR-108-111), Ministry of Science and Technology, Taiwan (Grant no. MOST108-2410-H-039-001 and MOST109-2410-H-039-001), and China Medical University (Grant no. CMU109-MF-119)"

***4****.We note that you have provided funding information that is not currently declared in your Funding Statement. However, funding information should not appear in the Acknowledgments section or other areas of your manuscript. We will only publish funding information present in the Funding Statement section of the online submission form.*

*Please remove any funding-related text from the manuscript and let us know how you would like to update your Funding Statement. Currently, your Funding Statement reads as follows:*

*"NO - Include this sentence at the end of your statement: The funders had no role in study design, data collection and analysis, decision to publish, or preparation of the manuscript"*

*Please include your amended statements within your cover letter; we will change the online submission form on your behalf.*

**Reply:** Thank you for the important comment. We have deleted the statement entirely as suggested.

***5.*** *We note that you have indicated that data from this study are available upon request. PLOS only allows data to be available upon request if there are legal or ethical restrictions on sharing data publicly. For information on unacceptable data access restrictions, please see http://journals.plos.org/plosone/s/data-availability#loc-unacceptable-data-access-restrictions.*

*In your revised cover letter, please address the following prompts:*

*a) If there are ethical or legal restrictions on sharing a de-identified data set, please explain them in detail (e.g., data contain potentially identifying or sensitive patient information) and who has imposed them (e.g., an ethics committee). Please also provide contact information for a data access committee, ethics committee, or other institutional body to which data requests may be sent.*

**Reply:** We thank for this important comment.The anonymized and de-identified data used in this study was provided by the *Health and Welfare Data Science Center* (HWDC), Ministry of Health and Welfare in Taiwan. When the researchers filled out the data access application form, the HWDC regulations stated that the data sharing is not allowed because of the concern that data may contain potentially identifying or sensitive patient information. Only the statistical outputs can be brought out of the HWDC. Therefore, the researchers cannot share the study data with anyone. However, all relevant data are within the paper.

The contact information of the HWDC((Health and Welfare Data Science Center) is as follows:

stsung@mohw.gov.tw

stcharlesfeng@mohw.gov.tw

https://dep.mohw.gov.tw/dos/cp-2516-59203-113.html

Health and Welfare Data Science Center, Ministry of Health and Welfare, Taiwan

The ethics committee related to this study:

irb@mail.cmuh.org.tw

http://web.cmuh.cmu.edu.tw/2007/IRB/index.html

Research Ethics Committee of China Medical University and Hospital, Taichung, Taiwan

**6.** *If there are no restrictions, please upload the minimal anonymized data set necessary to replicate your study findings as either Supporting Information files or to a stable, public repository and provide us with the relevant URLs, DOIs, or accession numbers. Please see http://www.bmj.com/content/340/bmj.c181.long for guidelines on how to de-identify and prepare clinical data for publication. For a list of acceptable repositories, please see http://journals.plos.org/plosone/s/data-availability#loc-recommended-repositories.*

*We will update your Data Availability statement on your behalf to reflect the information you provide.*

**Reply:** We thank for the comment. The de-identified data used in this study must be accessed and analyzed only in the HWDC(Health and Welfare Data Science Center) under the prohibition of data sharing. All relevant data are within the paper.

***7.****Please amend your list of authors on the manuscript to ensure that each author is linked to an affiliation. Authors’ affiliations should reflect the institution where the work was done (if authors moved subsequently, you can also list the new affiliation stating “current affiliation:….” as necessary).*

**Reply:** Thanks for the suggestion. We have updated the list of authors.

***8.*** *Please amend either the abstract on the online submission form (via Edit Submission) or the abstract in the manuscript so that they are identical.*

**Reply:** Thank you for the comments. As suggested, we have revised the abstract accordingly. The abstracts are now identical.

**Response to Reviewer**

**Reviewer #1**
**#** *Although the research could potentially fill an existing knowledge gap, the provided data do not support the conclusion. First of all, the distribution of the stroke risk factors in case group and control group is not equal. Adjusting the stated risk factors would not solve the problem of confounding, as the potential confounders are more than those suggested by authors.* *Secondly and related to the first point, the risk of confounding by the indication is likely for observed results. Many details that could clarify the issue are missing, including the type of used NSAIDS, the dose (not only the duration) of used NSAIDs, the time between NSAID consumption and incidence of stroke, and so on. Moreover, for those who experienced stroke in the case group (and majority fell in the group of using more than 24 days/month) the cumulative used NSAID is unclear.*

**Reply:** Thank you for the suggestion. This study was intended to evaluate NSAIDs use associated with women with dysmenorrhea develop stroke. For developing the study groups, therefore, we first identified women with dysmenorrhea excluding those with history of hysterectomy or ovariectomy and stroke. We searched for comorbidities that were potentially linked to stroke risk, including diabetes, hypertension, hyperlipidemia, obesity, arrhythmia, thyroid disease and alcohol-related diseases. Data analysis involved the use of logistic regression analysis as the statistical method. We cannot get the dose data used in the medical insurance database, we can calculate the number of days she has used it. In the revision, we addressed the issue of lacking the type of used NSAIDS, the dose of used NSAIDs, the time between NSAID consumption as a study limitation.

We have re-write the limitation section as “Although this study was strengthened by the use of the large insurance claims database, there were several limitations. First, information on body mass index, lifestyle of diet, smoking and drinking, and family health history was unavailable to adjust for these potential confounders in data analyses. However, the impact from some of these factors might be minor because women are more likely to avoid unhealthy behaviors. Smoking and drinking are rare habits in women in Taiwan and obesity is not prevalent as well. The study results might not be generalizable to non-Chinese populations and populations with higher rates of obesity. Second, the issue of lacking the type of used NSAIDS, the dose of used NSAIDs, the time between NSAID consumption which might increase the stroke in these groups.”

**#***The authors also do not provide any clue about the type of observed stroke in the cases and controls, whereby the plausibility of the conclusion could be improved. Another problem that may violate the conclusion of the study is the access to OTC NSAIDs which is totally neglected.*

**Reply:** Thank you for the comment and suggestion. We have added the diagnosis of stroke in data analysis of methodology section. Stroke types include hemorrhagic stroke (HS, ICD-9 codes 430-432), ischemic stroke (IS, ICD-9 code 434), transient cerebral ischemia (TIA, ICD-9 code 435) and other acute and other ill-defined cerebrovascular diseases (OCD, ICD-9 code 436 and 437). Because NSAIDs as common general use OTC medications in the Taiwan society and easy to buy at pharmacies. We have added this limitation in the context. “Third, NSAIDs as common general use OTC medications in the Taiwan society and easy to buy at pharmacies. However, with this lack of involvement of qualified healthcare professionals (example ;Doctor、Pharmacist) in the use of NSAIDs treatment of dysmenorrhea.”

***#****Since there are large scale meta-analyses that concluded no association between NSAIDs and stroke, in general, the authors could argue those findings only and only with providing the detailed information above to be able to reject the null hypothesis with a low risk of type one error. The statistical analysis is not appropriately done. The used statistical software is not stated, as well as the specific statistical tests the authors applied to the data. Where overall means or proportions could be simply compared, those comparisons are missing.*

**Reply:** Thank you for the comment and suggestion. Recently some studies investigating the incidence of adverse cardiovascular risk s associated with the administration of NSAIDs. This risk of was found to increase with higher daily doses and longer duration of use of NSAIDs. Stimulate our interest in using big data to investigate association between NSAIDs and stroke. We use the data from the Longitudinal Health Insurance Database 2000 (LHID2000) of the National Health Research Institutes, which is a subset of insurance claims from one million individuals randomly selected from 23.4 million insured people in Taiwan.

We added the used statistical software in the context.

We have re-organized the whole Methodology section in the revision. We briefly interpreted selection criteria then organized with subheadings: “Study design and study population”, “Selection Criteria”, “Stroke outcome and associated factors”, and data analysis. We have re-write the paragraph of selection criteria and added the stroke outcome and associated factors. we have further analyzed logistic regression analysis was used to calculate the crude and adjusted odds ratios (cOR and aOR) of stroke and the 95% confidence intervals (CI) to identify whether these variables were also associated with NSAIDs user. (Please see he revised manuscript. Table 1 is also presented next page in this reply.)

**Table 1: Baseline comparison in women with dysmenorrhea, with and without NSAIDs uses**

|  | NSAID users | | | |  |  |  |
| --- | --- | --- | --- | --- | --- | --- | --- |
|  | Yes | | No | | p-value | Odds ratio(95%CI) |  |
|  | N=3238  (13.0%) | | N=21717  (87.0%) | |  |  |  |
| Age, years | n | (%) | n | (%) | 0.009 | Crude | Adjusted |
| 15-19 | 345 | (10.7) | 2703 | (12.5) |  | Ref. |  |
| 20-24 | 690 | (21.3) | 4508 | (20.8) |  | 1.20 (1.05-1.38)** | 1.15 (1.00-1.32) |
| 25-29 | 726 | (22.4) | 4950 | (22.8) |  | 1.15 (1.00-1.32)* | 1.03 (0.90-1.19) |
| 30-39 | 945 | (29.2) | 6360 | (29.3) |  | 1.16 (1.02-1.33)* | 0.96 (0.83-1.10) |
| 40-49 | 532 | (16.4) | 3196 | (14.7) |  | 1.30 (1.13-1.51)*** | 1.01 (0.87-1.18) |
| Mean (SD) | 30.2 | (8.58) | 29.7 | (8.57) | 0.001 |  | 1.00 (0.99-1.00) |
| Follow-up years |  |  |  |  |  |  |  |
| Mean (SD) | 6.53 | (3.06) | 4.75 | (3.08) | <0.0001 |  |  |
| Pregnancy |  |  |  |  | <0.0001 |  |  |
| Ever | 1386 | (42.8) | 8336 | (38.4) |  | 1.20 (1.12-1.30)*** | 1.17 (1.08-1.27)*** |
| No | 1852 | (57.2) | 13381 | (61.6) |  | Ref. | Ref. |
| Comorbidity |  |  |  |  |  |  |  |
| Diabetes | 123 | (3.80) | 493 | (2.27) | <0.0001 | 1.70 (1.39-2.08)*** | 1.26 (1.01-1.57)* |
| Hypertension | 144 | (4.45) | 605 | (2.79) | <0.0001 | 1.62 (1.35-1.96)*** | 1.30 (1.06-1.58)* |
| Hyperlipidemia | 218 | (6.73) | 895 | (4.12) | <0.0001 | 1.68 (1.44-1.96)*** | 1.36 (1.15-1.62)*** |
| Obesity | 41 | (1.27) | 160 | (0.74) | 0.002 | 1.73 (1.22-2.44)** | 1.36 (0.95-1.93) |
| Arrhythmia | 229 | (7.07) | 953 | (4.39) | <0.0001 | 1.66 (1.43-1.93)*** | 1.46 (1.26-1.71)*** |
| Thyroid disease | 416 | (12.9) | 1988 | (9.15) | <0.0001 | 1.46 (1.31-1.64)*** | 1.32 (1.18-1.48)*** |
| Alcohol-related diseases | 48 | (1.48) | 165 | (0.76) | <0.0001 | 1.97 (1.42-2.72)*** | 1.76 (1.27-2.45)*** |
| NSAIDs: Nonsteroidal anti-inflammatory drugs  Adjusted OR: multiple analysis after adjusting for age, pregnancy, comorbidities, and medication; CI, confidence interval.  * p <0.05, ** p <0.01, *** p <0.001 | | | | | | | |

***#****The English should be improved, as there are a lot of grammatical defects.*

**Reply:** Thank you for the comment and suggestion. We have proofread and corrected the manuscript. This manuscript has proofread by a native English speaker prior to submission. We have provided the editing supporting document.

**Response to Reviewer**

**Reviewer #2**

**#***In the selection criteria, The author did not mention the causes of dysmenorrhea on the basis of which they conducted this study.*

**Reply:** Thank you for the comment. We have re-organized the whole Methodology section in the revision. We briefly interpreted selection criteria then organized with subheadings: “Study design and study population”, “Selection Criteria”, “Stroke outcome and associated factors”, and data analysis. We have re-write the paragraph of selection criteria and added the stroke outcome and associated factors.

**#***The Authors didn't mention about how long the patients used the NSAIDs? How does taking NSAIDs twice can cause a stroke?*

**Reply:** Thank you for the comment and suggestion. The case group included subjects who had a documented history of using NSAIDs for the treatments of dysmenorrhea, at least twice. It can be confirmed that he is using this drug. The issue of lacking the type of used NSAIDS, the dose of used NSAIDs, the time between NSAID consumption which might increase the stroke in these groups. We have added this in the third limitation in the context.

**Response to Reviewer**

**Reviewer #3**

Good article. Consider to reword some of the discussion to ensure the audience able to follow. Grammar and typos need correction. Double brackets are confusing so please change. Ensure that the conclusion in abstract tally with the discussion overall. However, authors need to highlight the main findings for the study.

**1.** *Good article with a lot of data presented in the manuscript.*

**Reply:** Thank you for the comment.

**2.** *Abstract: results section: statement is too generalised. Suggest to reword to highlight the important results*

**Reply:** Thank you for the comment and suggestion. We have revised result as “

Age and use of NSAIDs and its duration of usage were factors associated with an increased incidence of stroke. The stroke incidence rate was 12.77 per 10,000 person-years, and 1.83-fold higher in NSAIDs use cohort than in comparisons with adjusted hazard ratio (aHR) of 1.47 (95% CI=0.93-2.32). Among women with dysmenorrhea use of NSAIDs, the stroke incidence increased to 2.29-fold ( aHR 95% CI=1.36-3.84) in those use for ≧24 days per month and to 0.51-fold ( aHR 95% CI=0.13-2.10) in those use for 6-12 days per month.”

**3**. *Abstract: conclusion does not reflect the objective of the study, consider to emphasize the main ideas*

**Reply:** Thank you for the comment and suggestion. We have revised conclusion as “Women with dysmenorrhea who use NSAIDs have a higher risk of stroke. Especially young women, the risk of stroke is further increased, and the longer the medication, the higher the risk of stroke. Every woman with symptoms of dysmenorrhea deserves specialized outpatient treatment and care.”

**4.** *Page 8 : Statement ‘ the study results indicated…..’ suggest to put in the discussion*

**Reply:** Thank you for the comment and suggestion. We have moved this sentence to the discussion section.

**5.** *Figure 1 comparing 70(0.32%) controls vs 27 (0.83%) cases. Please indicate the p value*

**Reply:** Thank you for the inspirational comment and suggestion. We have added the p value in the Figure1.(Please see Figure1 in the revised manuscript)

**6.** *The authors suggest that NSAIDs have a high risk for stroke. However, the table 1 showed s those on NSAIDs also have other comorbidities (diabetes, hypertension etc) which are the traditional risk factors for stroke. How do the authors taken this into account? Arrhythmia, thyroid disease, hyperlipidemia accounted for the high number of cases. However, need to specify if it was atrial fibrillation as this will cause high incidence of embolic stroke*

**Reply:** Thank you for the comment and suggestion. We searched for comorbidities that were potentially linked to stroke risk, including Diabetes (ICD-9 code:250),

dysmenorrhea	(ICD-9 code: 625.3), Hypertension(ICD-9 code: 401-405), Hyperlipidemia(ICD-9 code: 272), Obesity(ICD-9 code: 278), Arrhythmia(ICD-9 code: 427),Thyroid disease(ICD-9 code: 240-246 ),Alcohol-related diseases(ICD-9 code: 291, 3030, 305, 571.0-571.3, 790.3, V11.3 ). The Non-specific code 427 Cardiac dysrhythmias include paroxysmal ventricular tachycardia convert, atrial fibrillation and flutter, ventricular fibrillation and flutter, cardiac arrest, premature beats,

other specified cardiac dysrhythmias and sinoatrial node dysfunction convert.

**7.** *Authors did not specify what type of stroke in the article? Infarct or haemorrhage ? How was the diagnosis of stroke made?*

**Reply:** Thank you for the comment and suggestion. We have added the diagnosis of stroke in data analysis of methodology section. Stroke types include hemorrhagic stroke (HS, ICD-9 codes 430-432), ischemic stroke (IS, ICD-9 code 434), transient cerebral ischemia (TIA, ICD-9 code 435) and other acute and other ill-defined cerebrovascular diseases (OCD, ICD-9 code 436 and 437).

**8*.*** *In the text, certain words like ‘women with dysmenorrhea’ are in upper case. Please change to lower case. Double brackets in the results are confusing, suggest to change,*

**Reply:** Thank you for the comment and suggestion. Our apologies for the type error. We have made correction in the revised manuscript.

(Please see Page7 page10 ,11,12,14 in the revised manuscript.)

**9.** *Discussion should highlight on the study’s main findings regarding NSAIDs use and stroke risk*

**Reply:** Thank you for the comment and suggestion. We added the main finding in the discussion section in first paragraph.

**10.** *Table 2 need to simplify to allow easy understanding*

**Reply:** Thank you for your suggestion for reformatting the 2 Tables. For the new Table 2 , we stratified by age group divided into comparison cohort and NSAIDs use cohort. as symbol We also added the symbol “ †” in the event less than 5.

(Please see he revised manuscript. Table 2 is also presented next page in this reply.)

| **Table 2. Risk of stroke compared between NSAIDs use cohort and comparison cohort among women with dysmenorrhea** | | | | | | | |  | |
| --- | --- | --- | --- | --- | --- | --- | --- | --- | --- |
| Age, years |  | Event | PY | IR | HR (95% CI) | | | | |
|  | Cohort |  |  |  | Crude | p | Adjusted* | | p |
| 15-19 | Comparison | 3**†** | 16667 | 1.80 | 1.0 |  | 1.0 | |  |
|  | NSAIDs | 2**†** | 2581 | 7.75 | 4.81 (0.80-28.8) | 0.09 | 5.23 (0.87-31.3) | | 0.07 |
| 20-24 | Comparison | 5 | 23965 | 2.09 | 1.0 |  | 1.0 | |  |
|  | NSAIDs | 4**†** | 4749 | 8.42 | 3.95 (1.05-14.8) | 0.04 | 4.27 (1.13-16.1) | | 0.03 |
| 25-29 | Comparison | 8 | 22186 | 3.61 | 1.0 |  | 1.0 | |  |
|  | NSAIDs | 0 | 4727 | 0.00 | NA |  | NA | |  |
| 30-39 | Comparison | 25 | 26731 | 9.35 | 1.0 |  | 1.0 | |  |
|  | NSAIDs | 14 | 5754 | 24.33 | 2.46 (1.27-4.76) | 0.008 | 2.22 (1.13-4.36) | | 0.02 |
| 40-49 | Comparison | 29 | 13510 | 21.47 | 1.0 |  | 1.0 | |  |
|  | NSAIDs | 7 | 3327 | 21.04 | 0.91 (0.40-2.10) | 0.83 | 0.70 (0.30-1.62) | | 0.40 |
| All | Comparison | 70 | 103058 | 6.79 | 1.0 |  | 1.0 | |  |
|  | NSAIDs | 27 | 21138 | 12.77 | 1.83 (1.17-2.86) | 0.008 | 1.47 (0.93-2.32) | | 0.10 |
| PY: person-years; IR: incidence rate per 10000 PY; HR: hazard ratio; CI: confidence interval; | | | | | | | |  | |
| *Model was adjusted for age, pregnancy and all comorbidities and medications listed in Table 1  **† event: less than 5** | | | | | | | |  | |

| 11. *Table 3 – less than 5, use the symbol*    Reply: Thank you for the comment and suggestion. We have added the event less than 5 as symbol ” ^†^ “ in the Table3.  Table 3 is also presented next page in this reply.  **Table 3:** Stroke risk seen in women with dysmenorrhea in association with use of NSAIDs and its duration of usage | | | | | | | | |
| --- | --- | --- | --- | --- | --- | --- | --- | --- |
|  | N | Event | PY | IR | HR (95% CI) | | | |
|  |  |  |  |  | Crude | p | Adjusted* | p |
| NSAIDs use day, per month |  |  |  |  |  |  |  |  |
| None | 21717 | 70 | 103058 | 6.79 | 1.00 |  | 1.00 |  |
| <=5 | 230 | 0 | 2006 | 0.00 | NA |  | NA |  |
| 6-12 | 763 | 2 ^†^ | 5472 | 3.65 | 0.52 (0.13-2.13) | 0.36 | 0.51 (0.13-2.10) | 0.35 |
| 13-24 | 851 | 5 ^†^ | 5581 | 8.96 | 1.30 (0.52-3.21) | 0.58 | 1.17 (0.47-2.91) | 0.73 |
| ≧24 | 1394 | 20 | 8079 | 24.76 | 3.56 (2.16-5.86) | <0.0001 | 2.29 (1.36-3.84) | 0.002 |
| PY: person-years; IR: incidence rate per 10000 PY; HR: hazard ratio; CI: confidence interval;  *Model was adjusted for age, pregnancy and all comorbidities listed in Table 1.  ^†^event: less than 5 | | | | | | | | |

**12.** *References are not standardised. Books and journals*, 7,8,17,20,21,23,24,25,27,28,30,31

**Reply:** Thank you for the comment and suggestion. We have proofread and corrected the all references format. Please see the revised manuscript.
